# Impact of the transpulmonary pressure on right ventricle impairment incidence during acute respiratory distress syndrome: a pilot study in adults and children

**DOI:** 10.1186/s40635-024-00671-2

**Published:** 2024-09-27

**Authors:** Meryl Vedrenne-Cloquet, Matthieu Petit, Sonia Khirani, Cyril Charron, Diala Khraiche, Elena Panaioli, Mustafa Habib, Sylvain Renolleau, Brigitte Fauroux, Antoine Vieillard-Baron

**Affiliations:** 1 Service de Réanimation et Surveillance Continue Médicochirurgicale Pédiatrique, Necker Hospital, APHP, 149 Rue de Sèvres, 75015 Paris, France; 2 Université Paris Cité, Paris, France; 3 Unité de Ventilation Non Invasive et du Sommeil de l’enfant, EA7330 VIFASOM, Université Paris Cité, Paris, France; 4 Medical Intensive Care Unit, Ambroise Paré Hospital, APHP, Boulogne, France; 5 INSERM UMR 1018, Clinical Epidemiology Team, CESP, Université de Paris Saclay, Villejuif, France; 6 ASV Santé, Genevilliers, France; 7 Service de Cardiologie Pédiatrique, M3C-Necker, Necker Hospital, APHP, Paris, France

**Keywords:** Right ventricle failure, Acute cor pulmonale, Transpulmonary pressure, Lung stress, Acute respiratory distress syndrome, Pediatric acute respiratory distress syndrome

## Abstract

**Background:**

Right ventricle impairment (RVI) is common during acute respiratory distress syndrome (ARDS) in adults and children, possibly mediated by the level of transpulmonary pressure (*P*_L_). We sought to investigate the impact of the level of *P*_L_ on ARDS-associated right ventricle impairment (RVI).

**Methods:**

Adults and children (> 72 h of life) were included in this two centers prospective study if they were ventilated for a new-onset ARDS or pediatric ARDS, without spontaneous breathing and contra-indication to esophageal catheter. Serial measures of static lung, chest wall, and respiratory mechanics were coupled to critical care echocardiography (CCE) for 3 days. Mixed-effect logistic regression models tested the impact of lung stress (Δ*P*_L_) along with age, lung injury severity, and carbon dioxide partial pressure, on RVI using two definitions: acute cor pulmonale (ACP), and RV dysfunction (RVD). ACP was defined as a dilated RV with septal dyskinesia; RVD was defined as a composite criterion using tricuspid annular plane systolic excursion, S wave velocity, and fractional area change.

**Results:**

46 patients were included (16 children, 30 adults) with 106 CCE (median of 2 CCE/patient). At day one, 19% of adults and 4/7 children > 1 year exhibited ACP, while 59% of adults and 44% of children exhibited RVD. In the entire population, ACP was present on 17/75 (23%) CCE. ACP was associated with an increased lung stress (mean Δ*P*_L_ of 16.2 ± 6.6 cmH_2_O in ACP vs 11.3 ± 3.6 cmH_2_O, adjusted OR of 1.33, CI95% [1.11–1.59], *p* = 0.002) and being a child. RVD was present in 59/102 (58%) CCE and associated with lung stress. In children > 1 year, PEEP was significantly lower in case of ACP (9.3 [8.6; 10.0] cmH_2_O in ACP vs 15.0 [11.9; 16.3] cmH_2_O, *p* = 0.03).

**Conclusion:**

Lung stress was associated with RVI in adults and children with ARDS, children being particularly susceptible to RVI.

*Trial registration* Clinical trials identifier: NCT0418467.

**Supplementary Information:**

The online version contains supplementary material available at 10.1186/s40635-024-00671-2.

## Background

Right ventricle impairment (RVI) is common during adult [1] and pediatric acute respiratory distress syndrome (ARDS) [2]. The underlying mechanisms of RVI depends on the transpulmonary pressure (*P*_L_), which increases pulmonary vascular resistances (PVR) and RV afterload when excessive [3]. *P*_L_ reflects the distending pressure and stress of the lungs [4]. *P*_L_ is approximated using esophageal manometry as it is calculated as the difference between the airway pressure (*P*_AW_) and the esophageal pressure (*P*_ES_) used as a surrogate for pleural pressure [5]. In adults with ARDS, *P*_L_-guided strategies failed to improve patient’s outcome [6]. This may be partly explained by the lack of consideration of the impact of lung stress induced by ventilator settings on RV function. RVI has been reported to be associated with worse outcome. The risk factors for RVI include a lower PaO_2_/FiO_2_, a higher PaCO_2_, and a higher airway driving pressure (Δ*P*_AW_) [1]. However, no study has tested the potential impact of *P*_L_ on RV function in ARDS despite a strong pathophysiological rationale [7, 8]. In particular, children may be more susceptible to RVI because of their high PVR [9], their limited cardiac response to an increase in preload, and their high ventricle interdependence [10].

In this pilot study, we sought to evaluate the impact of *P*_L_ on the occurrence of ARDS-associated RVI in adults and children testing two different definitions of RVI.

## Methods

This pilot physiological study was conducted in two tertiary university hospitals in France: one pediatric intensive care unit (Necker universitary hospital, Paris) and one adult intensive care unit (Ambroise Paré universitary hospital, Boulogne-Billancourt). The study was approved by an institutional review board (CPP Sud-Ouest et Outre-Mer 3-n°2019-A02814-53) and registered on the clinicaltrials database (NCT 04184674). Written informed consent from the patient or their guardians was obtained before inclusion.

### Population

Patients suffering from new-onset (within 48 h following admission) ARDS [11, 12] were included if invasively ventilated. Exclusion criteria comprised: neonates ≤ 37 weeks of corrected gestational age and/or less than 72 h of age, pregnant women, contra-indication to esophageal catheter, extracorporeal membrane oxygenation, congenital heart defect affecting RV function, and lack of social coverage.

### Study procedure

Patients received ventilation using cuffed endotracheal tubes connected to an ICU ventilator (V500 or Evita XL, Draeger, Netherlands, for children. Hamilton S1, USA, for adults). The ventilator settings and adjuvant therapies were left at the physician’s discretion, following guidelines for lung-protective ventilation [11, 13, 14]. Briefly, the ventilation strategies used in both units were defined to deliver a tidal volume around 6 mL/kg of predicted body weight, with a maximal airway plateau pressure at 28–30 cmH_2_O, and a minimal positive end-expiratory pressure (PEEP) level set at 5 cmH_2_O, then adapted depending on severity and respiratory mechanics. Patients could be ventilated in volumetric or barometric mode, although volume-(assist) control ventilation was prioritized in the two centers. Esophageal pressure monitoring was not considered for ventilator settings modifications. Critical care echocardiography (CCE), followed by static respiratory mechanics measurements were performed at day one, then, in case of PEEP level modification, up to 3 days.

### Critical care echocardiography

CCE (transthoracic echocardiography for all, followed by transesophageal echography in adults) was performed by the same investigator along the procedure, according to a standard protocol [15]. RVI was defined as either acute cor pulmonale (ACP) or RV systolic dysfunction (RVD). The former was defined as a dilated RV (RV/left ventricle (LV) end-diastolic area ratio > 0.6) associated with a septal dyskinesia, [16] and the latter as a fractional area change (FAC) < 35%, and/or a tricuspid annular plane systolic excursion (TAPSE) < 16 mm in adults or a z-score < − 2 in children [17], and/or a S wave velocity < 10 cm/s [18]. Each CCE could be classified as ACP only, RVD only, or both ACP and RVD. All the ultrasound images and loops were stored and exported to dedicated software (Echopac™, version 201, GE Healthcare Systems, Chicago, USA). A blinded analysis was performed off-line (Echopac) by two cardiopediatricians for children, and two intensivists for adults, all having expertise in RV assessment.

### Respiratory and lung mechanics

*P*_ES_ was measured using an esophageal balloon catheter (Marquat, France, for children; Nutrivent, Italy, for adults) inserted as recommended [19, 20], and filled with the appropriate volume of air [21, 22]. Airflow and airway pressure (*P*_AW_) were measured using a proximal calibrated pneumotachometer (Hans Rudolph, USA, for children; Hamilton, USA, for adults). All signals were displayed on the ventilator screen in adults, and recorded at 200 Hz using an analogical/numeric acquisition system (MP150, Biopac systems, USA) run on a PC computer and displayed using dedicated software (AcqKnowledge, version 4.2, Biopac systems) in children. Tidal volume was obtained by integrating the airflow signal over time.

Gas leaks were carefully excluded before measurements. After one minute of stable breathing, the investigator performed 3 consecutive inspiratory and expiratory holds. Airway and esophageal plateau pressures were obtained at the end of the inspiratory hold, while airway and esophageal PEEP were obtained at the end of a prolonged expiratory hold (4 s in adults, 3 in children) to account for intrinsic PEEP [23]. *P*_L_ was calculated online by subtracting *P*_ES_ from *P*_AW_. The subsequent transpulmonary driving pressure (Δ*P*_L_) and compliances of the respiratory system (*C*_RS_), lung (*C*_L_), and chest wall (*C*_CW_) were calculated and normalized on actual (children) or predicted (adults) body weight. Expiratory-*P*_L_ (*P*_L-EXP_) was computed directly from the measured end-expiratory *P*_L_, while inspiratory-*P*_L_ (*P*_L-INSP_) was computed using the elastance ratio method [24].

### Analyses

CCE data were first described at *T*_0_ at the patient level according to the presence or not of RVI, in adults, and children. Each measure of respiratory mechanics was coupled to simultaneous CCE to assess the impact of *P*_L_ on RVI using mixed-effect logistic regression models (one for ACP, one for RVD). For each model, the patient was treated as a random effect, to adjust for the correlation between repeated measures within the same patient. Based on the ACP prevalence in adults, and the physiological differences between children and adults, we anticipated RVI prevalence of 30% in the total population. Assuming the inclusion of 45 patients with at least 2 measurements, 4 variables could be included in the models. Variables of interest were selected a priori based on the physiologic rationale and the current literature evidence regarding ARDS-associated RVI, with variables exhibiting collinearity excluded. Four variables of interest were finally included: Δ*P*_L_, partial pressure of carbon dioxide (pCO_2_), lung injury severity, and age [1, 25, 26]. Δ*P*_L_ was used as a continuous variable as a surrogate for the tidal lung stress. PCO_2_ was measured using arterial blood gas in adult patients, and in either arterial or venous blood gas in children. Because of the differences between pediatric and adult guidelines regarding the estimation of hypoxemia and lung injury severity, we considered the level of the arterial partial pressure of oxygen to fraction of inspired oxygen ratio (PaO_2_/FiO_2_) and the oxygenation index (OI) in children. OI was either measured or calculated from the oxygen saturation index (OSI) (using the equation OI = 0.0745 + 1.7830 * OSI [27]) in the absence of arterial blood gas. Lung-injury severity was then categorized in mild/moderate or severe ARDS following the recommended thresholds of PaO_2_/FiO_2_ (severe ARDS if PaO_2_/FiO_2_ ≤ 100) or OI (severe ARDS if OI ≥ 16) [12, 28]. This transformation of the variable allowed to minimize the number of missing data, and to achieve the greatest homogeneity in the population. Because of a highly skewed distribution and to distinguish adults and children, age was also categorized in two classes: adults (≥ 18 years old) and children (< 18 years old). Because echocardiographic assessment of RV dilation might be uncertain in infants [29], children less than 1 year old were excluded from all the analyses using the ACP definition.

Respiratory mechanics were then compared in CCE studies with or without RVI, in adults and children separately, using the t-test or Wilcoxon rank sum test depending on the variables’ normality. Continuous variables were presented as means ± standard deviations (SD) or medians and interquartile ranges (IQR) depending on the sample size and their distribution, and categorical variables as numbers and percentages. We also described the relationship between Δ*P*_L_ and driving airway pressure (Δ*P*_AW_) using linear regression model. Data were analyzed using R programming software. A *p* value < 0.05 defined statistical significance.

## Results

### Population

**Fig. 1:**
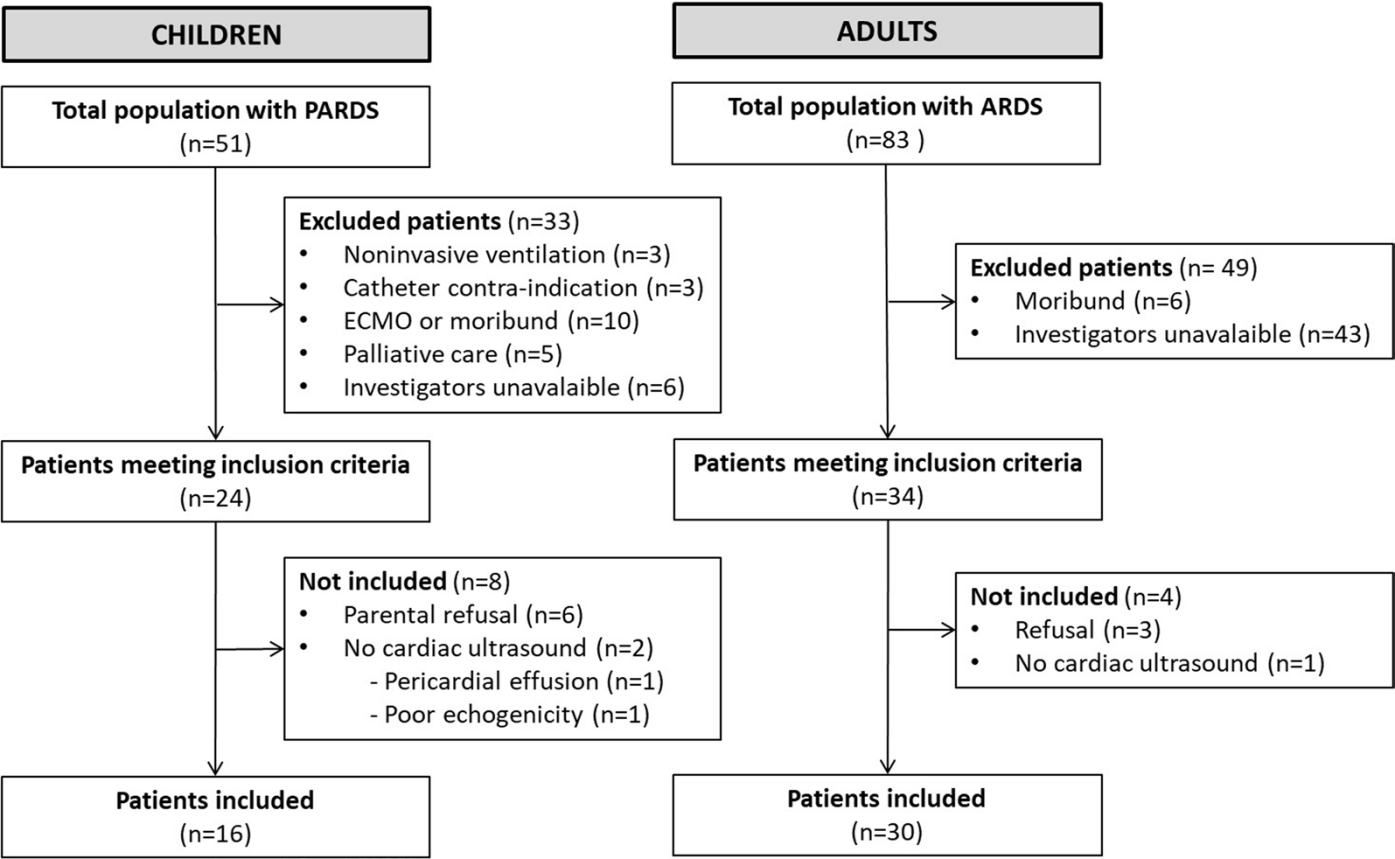
Flowchart

From July 2020 to July 2022, 83 adults and 51 children were admitted in the two centers for ARDS or PARDS. Among those, 58 patients met the inclusion criteria and 46 were included (16 children, 30 adults) (Fig. 1). Patients’ characteristics are presented in Table 1. Median [IQR] age was 0.7 [0.4; 7.1] years for children, and 67 [61; 72] years for adults. The most common trigger for ARDS was pneumonia, especially due to SARS-CoV2 infection in adults (80%). The majority of the patients had severe ARDS. Mortality rate in the intensive care unit at 28 days was 13% for children and 60% for adults (Table 1).

**Table 1:** Patients’ characteristics

	Children (*n* = 16)	Adults (*n* = 30)
Baseline characteristics		
Age (y)	0.7 [0.4; 7.1]	67 [61; 72]
Male/female, *n* (%)	8 (50)/8 (50)	18 (60)/12 (40)
Pulmonary ARDS, *n* (%)	15 (94)	29 (97)^a^
Mild/moderate/severe	3 (19)/3 (19)/10 (62)	0 (0)/6 (20)/24 (80)
Immune deficiency, *n* (%)	9 (56)	6 (20)
Obesity, *n* (%)	2 (13)	11 (37)
PELOD2 (children)/SOFA score (adults)	5 [3; 7]	7 [4; 8]
OSI	10.6 [8.3; 12.2]	11.5 [9.5; 14.6]
PaO_2_/FiO_2_	142 [121; 150]^c^	75 [58; 97]
PaCO_2_ (mmHg)	55 [50; 59]	47 [40; 51]
Ventilation		
Volume/pressure assisted control, *n* (%)	14 (88)/2 (12)	30 (100)/0 (0)
Preset *V*_T_ (mL/kg)	5.4 [4.7; 7.7]	5.8 [4.9; 6.7]
Preset Inspiratory pressure (cmH_2_O)	27 [27; 28]	
Preset PEEP (cmH_2_O)	8 [8; 10]	5 [5; 7]
RR (/min)	40 [31; 50]	18 [16; 22]
Hemodynamics		
Lactate (mmol/L)	1.5 [1.2; 1.8]	2.2 [1.9; 3.0]
Fluid balance (mL/48 h)^b^	− 94 [− 183; 467]	320 [− 500; 1873]
Vasopressive drugs, *n* (%)	5 (31)	11 (37)
Volume expansion, *n* (%)	4 (25)	11 (37)
NO, *n* (%)	1 (6)	1 (3)
Prone position in the 24 h, *n* (%)	4 (25)	11 (37)
Outcome		
ECMO requirement, *n* (%)	2 (3)	0 (0)
Invasive VFD at day-28	9 [0; 20]	0 [0; 0]
Noninvasive VFD at day-28	0 [0; 16]	0 [0; 0]
LOS (days)	24 [11; 62]	16 [11; 24]
Death at day-28, *n* (%)	2 (13)	18 (60)
Death at day-90, *n* (%)	4 (25)	20 (67)

Data are presented in medians [IQR] for quantitative variables and *n* (%) for qualitative variables

Obesity was defined by a BMI (body mass index) > 30 kg/m^2^ in adults, and above the 97th percentile for age and sex in children

ARDS, acute respiratory distress syndrome; ECMO, extracorporeal membranous oxygenation; LOS, length of stay; NO, inhaled azote monoxide; OSI, oxygen saturation index; PEEP, positive end-expiratory pressure; PELOD2, pediatric logistic organ dysfunction score 2; RR, respiratory rate; VFD, ventilator-free days; *V*_T_, tidal volume

^a^ 24/30 patients (80%) had ARDS due to SARS-CoV2 pneumonia

^b^ Missing data for 9 children

^c^ Missing data for 12 children

### CCE studies at day 1

There were 5 (19%) adult patients exhibiting ACP on their first CCE (*T*_0_) and 17 (59%) RVD. RV/LV end-diastolic area ratio, S wave, TAPSE, and FAC seemed affected in patients with ACP and RVD as compared to their counterpart. RV/LV end-diastolic area ratio was significantly higher in adults with either ACP or RVD as compared to those without RVI (*p* = 0.01), with a significant alteration of left ventricle output (lower aortic velocity time integral, *p* = 0.01) (Table 2).

**Table 2:** Description of hemodynamics and cardiac ultrasound in adults at day 1 according to RV outcomes

	ACP (*n* = 26)			RVD (*n* = 29)		
	Yes *n* = 5 (19%)	No *n* = 21 (81%)	p	Yes *n* = 17 (59%)	No *n* = 12 (41%)	*p*
Age (y)	60 [44; 67]	66 [62; 72]	ns	67 [62; 70]	65 [60; 72]	ns
HR (bpm)	98 [95; 102]^1^	82 [71; 89]	0.03	91 [71; 97]^1^	84 [74; 92]	ns
Lactate (mmol/L)	3.3 [2.8; 3.9]^1^	2.3 [1.9; 2.8]^1^	ns	2.4 [2.1; 3.2]^1^	2.2 [1.5; 2.5]^1^	ns
Fluid balance (mL/24 h)	998 [693; 1441]^1^	− 200 [− 520;1115]^1^	ns	626 [− 237;1441]^1^	− 164 [− 565; 895]	ns
Vasoactive drugs	3 (60)	5 (24)	ns	8 (47)	2 (17)	ns
RV/LV area	0.87 [0.69; 1.00]	0.60 [0.46; 0.68]	0.01	0.69 [0.64; 0.92]	0.51 [0.47;0.63]^1^	< 0.01
TAPSE (mm)	16 [13; 19]^2^	21 [19; 23]^1^	ns	18 [16; 21]^5^	22 [21; 24]^1^	0.01
S wave (cm/s)	14.5 [11.8; 15] ^1^	16 [14; 18]^2^	ns	15 [11; 17]^3^	16 [15; 18]^1^	ns
FAC (%)	18 [18; 30]	37 [21; 43]^2^	ns	21 [17; 31]	42 [37; 44]^3^	< 0.01
RVFWS	− 21 [− 24; − 18]^1^	− 27 [− 28; − 24]^11^	ns	− 22 [− 28; − 20]^9^	− 27 [− 28; − 26]^6^	ns
SPAP (mmHg)	31 [22; 34] ^2^	27 [22; 34]^8^	ns	31 [23; 33]^7^	27 [17; 33]^5^	ns
Aortic VTI	11 [9; 15]	18 [16; 21]	0.01	16 [14; 18]^1^	21 [19; 22]	< 0.01
CI (L/mn/m^2^)	1.7 [1.7; 1.9]^1^	2.1 [1.6; 2.7]	ns	1.7 [1.6; 2.3]^2^	2.7 [1.9; 3.4]	0.04
LVEF (%)	45 [39; 60]	58 [45; 65]	ns	45 [44; 65]	58 [52; 68]^1^	ns

Data are presented in medians [IQR] for quantitative variables and n (%) for qualitative variables. Numbers of missing values are indicated in exposure. Data compared using Wilcoxon test (continuous variables) or Fisher test (categorical variables)

ACP, acute cor pulmonale; FAC, fractional area change; HR, heart rate; LV, left ventricle; LVEF, left ventricular ejection fraction; RVD, right ventricular dysfunction; RV, right ventricle; RVFWS, RV free wall strain; SPAP, systolic pulmonary arterial pressure; TAPSE, tricuspid annular plane systolic excursion; VTI, velocity time integral; ns, non-significant (*p* ≥ 0.05)

Regarding children > 1 year of age, 7 had measurements of RV/LV end-diastolic area ratio and septal motion assessment, whose 4 had ACP. RV/LV end-diastolic area ratio, S wave, TAPSE z-score, and FAC seemed affected in children with ACP despite no statistical difference (Table 2). Seven (44%) children exhibited RVD on their first CCE. RVI did not impair LV systolic function in children (Table 3).

**Table 3:** Description of hemodynamics and cardiac ultrasound in children at day 1 according to RV outcomes

	ACP (*n* = 7)*			RVD (*n* = 16)		
	Yes *n* = 4 (57%)	No *n* = 3 (43%)	p	Yes *n* = 7 (44%)	No *n* = 9 (56%)	*p*
Age (y)	7.7 [5.8; 10.7]	11.9 [6.7; 12.5]	ns	0.6 [0.2; 6]	0.8 [0.5; 6.6]	ns
HR (bpm)	118 [105; 132]	138 [122; 139]	ns	134 [128; 155]	140 [132; 158]	ns
Lactate (mmol/L)	1.5 [1.3; 1.6]	1.8 [1.3; 1.9]	ns	1.7 [1.6; 2]	1 [0.8; 1.6]	0.03
Fluid balance (mL/24 h)	320 [314; 327]	477 [351; 604]	ns	− 110 [− 130; 112]^4^	308 [224; 600]^4^	ns
VIS	0 [0; 13]	0 [0; 0]	ns	0 [0; 15]	0 [0; 0]	ns
RV/LV area	1.00 [0.82; 1.29]	0.57 [0.57; 0.69]	ns	0.85 [0.72;1.18]^2^	0.82 [0.65; 1.21]	ns
TAPSE (mm)	17.2 [16.1; 19.0]	20.0 [17; 23.2]	ns	11 [11; 17]	14 [13; 16]	ns
z-score	− 1.5 [− 2.4; 0.0]	− 0.9 [− 1.0; 0.8]	ns	− 1.1 [− 2.4; − 0.5]	− 0.9 [− 0.9; − 0.6]	ns
S wave (cm/s)	13.5 [11.2; 15.2]	14.0 [13.0; 15.5]	ns	11 [9; 14]^1^	12 [11; 15]	ns
FAC (%)	36.5 [34.4; 44.9]	53 [48; 53]	ns	57 [39; 62]^1^	46 [43; 53]	ns
RVFWS	− 20 [− 21; − 19]	− 27 [− 31; − 22]^1^	ns	− 19 [− 20; − 18]^1^	− 21 [− 28; − 17]^1^	ns
SPAP (mmHg)	37 [35; 39]^2^	42 [42; 42]^1^	ns	41 [33; 51]^2^	51 [43; 57]^3^	ns
Aortic VTI	17 [16; 18]	22 [18; 23]	ns	17 [14; 18]	18 [15; 22]	ns
CI (L/mn/m^2^)	3.9 [3.1; 4.5]	4.4 [3.8; 4.9]	ns	3.8 [2.8; 4.4]	4.6 [3.8; 5.2]	ns
LVEF (%)	66 [62; 72]	67 [63; 71]	ns	70 [59; 73]	67 [61; 75]^1^	ns

Data are presented in medians [IQR] and compared using Wilcoxon tests. Numbers of missing values are indicated in exposure.*: children ≥ 1 year old

ACP, acute cor pulmonale; FAC, fractional area change; HR, heart rate; LV, left ventricle; LVEF, left ventricular ejection fraction; RVD, right ventricular dysfunction; RV, right ventricle; RVFWS, RV free wall strain; SPAP, systolic pulmonary arterial pressure; TAPSE, tricuspid annular plane systolic excursion; VIS, vasoactive inotrope score; VTI, velocity time integral; Ns, non-significant (p ≥ 0.05)

Among patients who benefitted from complete ultrasounds at day one, 25 had RVI with either CPA, RVF, or both (16 adults and 9 children): 9 adults (56%) and one child (11%) were deceased at day 28.

### Association between lung stress and RVI

Overall, 75 CCE studies were available in patients > 1 year of age to assess ACP, with 74 being coupled to respiratory mechanics measurements. ACP was present on 17/75 (23%) CCE. Lung stress was associated with the presence of ACP (mean Δ*P*_L_ of 16.2 ± 6.6 cmH_2_O in ACP vs 11.3 ± 3.6 cmH_2_O, adjusted OR of 1.33, CI95% [1.11–1.59], *p* = 0.002], whereas being an adult significantly decreased the risk of ACP (65% of adults in ACP vs 90%, adjusted OR of 0.09, CI95% [0.01; 0.62], *p* = 0.015). ARDS severity and the level of PCO_2_ were not associated with ACP (Table 4).

**Table 4:** Factors associated with right ventricle injury in patients with ARDS

	*n*	ACP *		Mixed-effect logistic regression		
		Yes *n* = 17 (23%)	No *n* = 58 (77%)	OR	CI95%	*p*
Age	75					
1–17 years		6 (35)	6 (10)			
≥ 18 years		11 (65)	52 (90)	0.09	0.01–0.62	0.015
ARDS severity	71					
Mild/moderate		6 (37)	19 (34)			
Severe		10 (63)	37 (66)	0.83	0.18–3.74	0.805
PaCO_2_ (mmHg)	71	51 ± 7	47 ± 9	0.98	0.90–1.08	0.717
∆*P*_L_ (cmH_2_O)	74	16.2 ± 6.6	11.3 ± 3.6	1.33	1.11–1.59	0.002

	*n*	RVD		Mixed-effect logistic regression		
		Yes *n* = 59 (58%)	No *n* = 43 (42%)	OR	CI95%	*p*
Age	102					
0–17 years		14 (24)	14 (33)			
≥ 18 years		45 (76)	29 (67)	2.06	0.44–9.67	0.359
ARDS severity	99					
Mild/moderate		22 (39)	16 (38)			
Severe		34 (61)	26 (62)	1.12	0.41–3.06	0.825
PaCO_2_ (mmHg)	94	49 ± 9	49 ± 9	0.99	0.93–1.05	0.743
∆*P*_L_ (cmH_2_O)	96	13.3 ± 5.3	11.4 ± 4.2	1.16	1.02–1.31	0.027

Data are presented in means ± SD for continuous variables or n(%) for categorical variables. * Patients > 1 year old

ACP, acute cor pulmonale; CI95%, confidence interval 95%; ARDS, acute respiratory distress syndrome; ∆*P*_L_, transpulmonary driving pressure; OR, odds ratio; RVD, right ventricle dysfunction

A total of 102 CCE studies were available to assess RVD, with 101 being coupled to respiratory mechanics measurements. RVD was observed in 59/102 (58%) CCE. Lung stress was associated with RVD (mean Δ*P*_L_ of 13.3 ± 5.3 cmH_2_O in RVD vs 11.4 ± 4.2 cmH_2_O, adjusted OR of 1.16, CI95% [1.02–1.31], *p* = 0.027). Age, ARDS severity, and the level of PCO_2_ were not associated with RVD (Table 4).

### Respiratory mechanics in adults and children with or without RVI

**Fig. 2:**
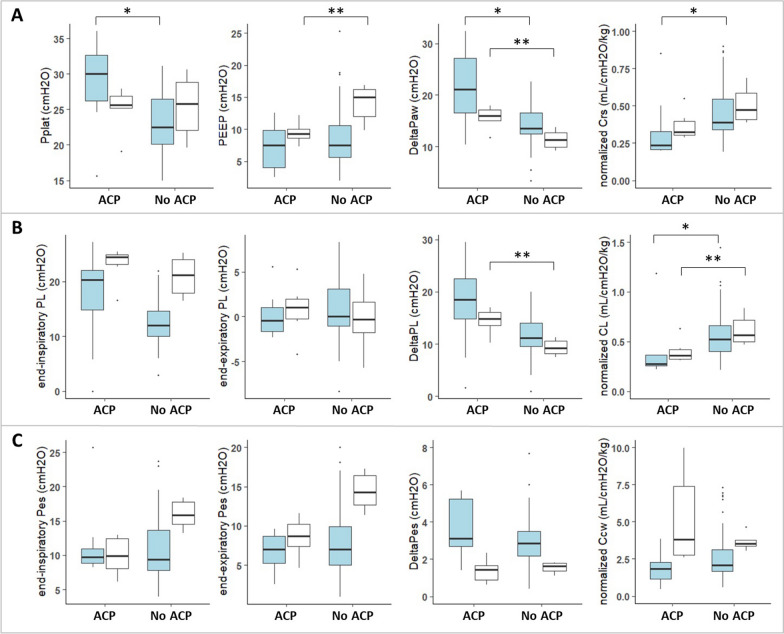
Comparison of respiratory mechanics in patients older than one year of age with or without acute cor pulmonale. Each available measure of respiratory mechanics was coupled to the simultaneous cardiac ultrasound. Data are presented for **A** respiratory, **B** lung, and **C** chest wall mechanics in ultrasounds of adults (blue boxes) and children (white boxes), with or without acute cor pulmonale. The central bars represent the medians; the lower and upper ends of the box represent, respectively, the 1st and 3rd quartiles; the T-bars represent the 10th and 90th percentiles; circles are outliers; extreme outliers are not represented in the figure (*n* = 2 for the normalized *C*_L_/kg, *C*_RS_/kg, and *C*_CW_/kg, *n* = 1 for Δ*P*_ES_). * *p* < 0.01 when ACP compared to no ACP; *p* < 0.05 when ACP compared to no ACP. ACP, acute cor pulmonale; *C*_CW_/kg, static compliance of the chest wall normalized per predicted body weight; *C*_L_/kg, static compliance of the lungs normalized per predicted body weight; *C*_RS_/kg, static compliance of the respiratory system normalized per predicted body weight; DeltaPaw, static airway driving pressure; DeltaPL, static transpulmonary driving pressure; DeltaPes, difference between end-inspiratory and end-expiratory oesophageal pressure; end-inspiratory Pes, value of oesophageal pressure at the end of the inspiratory hold; end-expiratory Pes, value of the oesophageal pressure at the end of the expiratory hold; end-inspiratory PL, end-inspiratory transpulmonary pressure (elastance-derived method); end-expiratory PL, end-expiratory transpulmonary pressure (direct method); PEEP, positive end-expiratory airway pressure; Pplat, airway plateau pressure

In CCE of adults, ACP was present in 10/62 (16%) CCE and significantly associated with both higher Δ*P*_AW_ (*p* = 0.01) and plateau *P*_AW_ (*p* < 0.001), and lower *C*_RS_ (*p* = 0.01) and *C*_L_ (*p* = 0.03), as compared to CCE without signs of ACP. Δ*P*_L_ was higher in case of ACP, without statistically significant difference (Fig. 2, online Table 1). RVD was associated with higher Δ*P*_L_ (*p* = 0.04) and plateau *P*_AW_ (*p* = 0.04) as compared to normal CCE (online Table 1).

In CCE of children, ACP was present in 6/12 (50%) CCE and significantly associated with higher Δ*P*_L_ (*p* = 0.002), Δ*P*_AW_ (*p* = 0.004), and elastance ratio (*E*_L_/*E*_RS_ 0.9 [0.89; 0.95] in CCE with ACP vs 0.83 [0.82–0.83], *p* = 0.002), and a lower normalized *C*_L_/kg (*p* = 0.02). PEEP was significantly lower in case of ACP (9.3 [8.6; 10.0] vs 15.0 [11.9; 16.3] cmH_2_O, *p* = 0.03) (Fig. 2, online Table 2). RVD was significantly associated with a higher tidal volume (*p* = 0.03) (online Table 2).

There was a strong linear relationship between Δ*P*_L_ and Δ*P*_AW_ in the entire population (*R* = 0.93, *p* < 0.01) (online Fig. 2).

## Discussion

To our knowledge, this physiological study is the first to investigate the complex relationship between transpulmonary pressure and RVI in a heterogeneous population of adults and children undergoing controlled ventilation for ARDS. We found that: (i) the level of Δ*P*_L_ as a surrogate for lung stress was associated with RVI, whatever the definition used; (ii) ACP seemed more common in children than adults; (iii) ACP was associated with lower PEEP levels in children.

Lung stress is one of the main determinants of mechanical ventilation-related injuries [30–32]. In clinical practice, lung stress is estimated by the plateau pressure and the driving airway pressure, which represents the best predictor of ACP [1] and mortality [25, 26] in adults with ARDS. However, the estimation of transpulmonary pressure using *P*_ES_ is the most precise marker of lung stress, and allows partitioning of lung and chest wall mechanics. With regard to the pathophysiology of RVI under mechanical ventilation, we used the Δ*P*_L_ as a surrogate for the tidal lung stress.

In adults, our results are in line with previous studies [1, 26]. Our results relative to the association between Δ*P*_AW_ and RVI is also consistent with the recent findings of Chen et al., who reported that the airway driving pressure was better associated with outcome than the driving transpulmonary pressure. Our observation may confirm that the airway driving pressure is a good indicator of ventilator-induced lung injury, but also contains some information about the patient’s severity, herein illustrated by RVI. In addition, the driving airway pressure was strongly correlated to Δ*P*_L_ in our population, suggesting that there is a potential benefit to use it as an estimate for lung stress in the most severe patients when esophageal pressure is not available. We believe, however, that Δ*P*_L_ represents the best physiological component to explain mechanical ventilation-related injuries, irrespective of patient severity. Partitioning lung and chest wall mechanics remains important to deliver not only a lung-protective, but also heart-protective tailored ventilation.

We cannot preclude the result we observed may be due to the overall severity in the adult population, as attested by the high mortality rate. We assume that this was due to the recruitment period during the beginning of SARS-CoV2 pandemic, with selected population of patients with SARS-CoV2 associated pneumoniae being intubated later than for usual ARDS. While ACP has been classically defined as the most severe form of RV failure, it is also crucial to detect early RV dysfunction. Our findings not only confirm the impact of high levels of lung stress on the presence of ACP, but also show that this relationship is present even in RVD, which represents a milder form of RVI.

The interpretation of respiratory mechanics measurements requires estimation of the airway occlusion pressure (AOP), which was not calculated in this study. Despite AOP being frequent in adults with ARDS, its prevalence is unknown in children because measures have not been validated. In addition, RVI may be related to either lung collapse or overinflation. AOP is always reached by inflation of the tidal volume in clinical practice. Then, closing pressure is probably more crucial to avoid lung collapse and is well-known to be much lower than the opening pressure [33]. In our study, especially in adults, PEEP was expected to avoid lung derecruitment and optimize compliance, and we did not find any intrinsic PEEP. Thus, we believe that the respiratory settings in adult patients might have limited the presence of AOP or its impact on driving pressure calculation. That being said, PEEP levels were relatively low in adults, with only 25% of them having PEEP above 7 cmH_2_O. We do not believe that PEEP levels would modify the relationship between lung stress and RVI, but setting a higher PEEP in patients with recruitable lungs may reduce both lung stress and RVI incidence, as we observed in our pediatric population.

Children have a more compliant chest wall and a smaller functional residual capacity, which may favor lung retraction and atelectasis. Interestingly, the PEEP level was higher in the absence of ACP and in case of lower lung stress. We may assume that in those cases, a higher PEEP level is associated with a better lung recruitment and improved lung compliance. Preliminary reports have recently showed the benefits of a *P*_L_-targeted PEEP titration on RV function in adults [34]. In this respect, setting a higher PEEP level in children with recruitable lungs may reduce *P*_L_ and its subsequent afterload effect, which may preserve RV function. We also observed a higher elastance ratio in children with ACP, which may suggest a higher transmission of the airway pressure to the lung in the most severe patients, as reported in adults [35]. Larger studies are needed to investigate how the elastance ratio differs in children as compared to adults.

There is a lack of evidence regarding RVI in critically ill children outside congenital heart defects, which may be partly explained because the ultrasound assessment of the RV is challenging, especially in the PICU [18, 36]. Thanks to our multimodal ultrasound analysis using two different definitions of RVI, we provided a detailed assessment of RV function along with respiratory mechanics in this poorly described population. RV dysfunction vary from 26 to 65% of children with ARDS according to the ultrasound parameter used [2]. Similarly, we found a high prevalence of both RVD and ACP in children. ACP has been scarcely studied in critically ill children because the accuracy and reliability of echocardiography assessment of RV size and of septal flattening are poor [29, 37]. In the youngest children who often face high PVR, RV might dilate in case of chronic pulmonary hypertension without RV congestion or dysfunction [38, 39]. In this situation, RV/LV area may reflect more the RV adaptation to afterload constraint than an accurate marker of RVI. We believe that intensivists should not focus on the RV/LV area ratio only to define ACP and RVI in neonates and infants.

Our study has limitations. First, the sample size was small, especially in children, and the non-inclusion of some patients due to logistic reasons may have introduced selection bias. As a consequence, we were not able to analyze the impact of lung stress according to PEEP levels. Second, pediatric and adult cardiac ultrasounds were analyzed separately by different investigators to maximize expertise, which may have induced an interpretation bias. However, we used homogenized definitions in both populations and performed blinded analyses. Third, *P*_ES_ gives only an estimation of *P*_L_ and its surrogates. Even if all measurements were performed as recommended, there are unique aspects regarding the type and placement of the catheter, as well as the volume of air inflated in the balloon to ensure accuracy, which are unique to children and still not validated. Finally, due to the recruitment period during the beginning of SARS-CoV2 pandemics, most adult patients had Covid-19 related ARDS, which leads to changes in vascularization likely to impact the relationship between lung stress and RVI [40].

## Conclusion

This original pilot study confirmed the impact of the lung stress on RV function during ARDS in non-spontaneously breathing adults and children, with the latter being particularly susceptible to RVI. How a ventilatory strategy based on the driving airway and transpulmonary pressures could prevent the occurrence of RVI and improve outcome remains to be demonstrated in both adult and pediatric settings.

## Supplementary Information

Supplementary Material 1.

## Data Availability

Data are available upon reasonable request from M.Vedrenne-Cloquet.

## Abbreviations

ACP: Acute cor pulmonale
ARDS: Acute respiratory distress syndrome
AOP: Airway occlusion pressure
CCE: Critical care echocardiography
*C*_RS_: Respiratory system compliance
*C*_L_: Lung compliance
FAC: Fractional area change
ICU: Intensive care unit
PARDS: Pediatric acute respiratory distress syndrome
RV: Right ventricle
RVFWS: Right ventricle free wall strain
RVI: Right ventricle impairment
RVD: Right ventricle dysfunction
STE: Speckle tracking echocardiography
TAPSE: Tricuspid annular plane systolic excursion
*P*_AW_: Airway pressure
*P*_ES_: Esophageal pressure
PICU: Pediatric intensive care unit
*P*_L_: Transpulmonary pressure
PVR: Pulmonary vascular resistances
