# Supplementary material for: Impact of the transpulmonary pressure on right ventricle impairment incidence during acute respiratory distress syndrome: a pilot study in adults and children
Source: Intensive Care Med Exp. 2024 Sep 27;12:84. doi: 10.1186/s40635-024-00671-2 (PMC11436589; doi:10.1186/s40635-024-00671-2)
Supplement: Supplementary file 1 — Supplementary Material 1. [file 40635_2024_671_MOESM1_ESM.docx]

**Impact of the Transpulmonary Pressure on right ventricle impairment incidence during Acute Respiratory Distress Syndrome: a pilot study in adults and children**

**Online table 1.** Comparison of respiratory mechanics in ultrasounds of adults with or without ARDS associated RV injury

|  | **ACP** | | | **RVD** | | |
| --- | --- | --- | --- | --- | --- | --- |
|  | **YES**  n=10 (16%) | **NO**  n=52 (84%) | p | **YES**  n=44 (60%) | **NO**  n=29 (40%) | p |
| **∆P_AW_ (cmH_2_O)** | 21.1 [16.5; 27.2] | 13.6 [12.5; 16.6] | **0.01** | 15.0 [12.7; 19.8] | 13.3 [12.4; 16.6] | ns |
| **Plateau P_AW_ (cmH_2_O)** | 30.0 [26.2; 32.7] | 22.5 [20.2; 26.5] | **<0.001** | 25.4 [21.5; 29.0] | 22 [20.2; 26.0] | **0.04** |
| **PEEP (cmH_2_O)** | 7.5 [4.0; 9.8] | 7.5 [5.6; 10.6] | ns | 7.4 [5.5; 10.5] | 7.4 [5.6; 9] | ns |
| **C_RS_ (mL/cmH_2_O)** | 23.8 [15.7; 32.8] | 35.2 [30.9; 39.5] | **0.01** | 33.5 [23,3; 38.2] | 35.6 [27.1; 38.8] | ns |
| **∆P_L_ (cmH_2_O)** | 18.5 [14.8; 22.6] | 11.2 [9.5; 14.1] | 0.05 | 12.5 [9.9; 17.6] | 10.8 [8.2; 14.6] | **0.04** |
| **P_L-INS_ (cmH_2_O)** | 20.4 [14.9; 22.0] | 12.0 [10.0; 14.6] | ns | 13.5 [10.1; 17.6] | 10.6 [9.4; 14.7] | ns |
| **P_L_-_EXP_ (cmH_2_O)** | -0.5 [-1.7; 1.0] | 0.0 [-1.1; 3.1] | ns | 0 [-1.6; 2.4] | 0.3 [-0.7; 3.2] | ns |
| **C_L_ (mL/cmH_2_O)** | 27.2 [19.6; 36.5] | 42.2 [34.9; 52.6] | **0.03** | 39.9 [27.9 ; 47.6] | 42.1 [34.8 ; 59.7] | ns |
| **C_CW_ (mL/cmH_2_O)** | 155 [137 ; 222] | 155 [84 ; 199] | ns | 170 [125 ; 215] | 153 [125 ; 217] | ns |
| **E_L_/E_RS_** | 0.85 [0.79 ; 0.89] | 0.82 [0.74 ; 0.87] | ns | 0.83 [0.78 ; 0.87] | 0.79 [0.69 ; 0.86] | ns |
| **VT/kg (mL/kg)** | 5.7 [5.4 ; 6.6] | 5.8 [4.8 ; 6.8] | ns | 5.9 [4.9 ; 6.8] | 5.8 [4.6 ; 6.9] | ns |

Each available measure of respiratory mechanics was coupled to the simultaneous cardiac ultrasound. Data are presented in medians [IQR] and compared using t-tests or wilcoxon rank sum tests depending of their normality.
* Exclusion of infants (<1 year).
Abbreviations: ACP: acute cor pulmonale; C_CW_: static compliance of the chest wall; C_L_: static compliance of the lungs; C_RS_: static compliance of the respiratory system; ∆P_AW_ : static airway driving pressure; ∆P_L_ : transpulmonary driving pressure; E_L_/E_RS_ : elastance ratio; P_L-INS_: end-inspiratory transpulmonary pressure (elastance-derived method); P_L-EXP_: end-expiratory transpulmonary pressure (direct method); Plateau P_AW_ : airway plateau pressure; PEEP: Positive end-expiratory airway pressure; Vt/kg: Tidal volume normalized per predicted body weight; RVD: Right ventricle dysfunction.

**Online table 2.** Comparison of respiratory mechanics in ultrasounds of children with or without ARDS associated RV injury

| **Variables** | **ACP *** | | | **RVD** | | |
| --- | --- | --- | --- | --- | --- | --- |
|  | **YES**  n=6 (50%) | **NO**  n=6 (50%) | p | **YES**  n=14 (50%) | **NO**  n=14 (50%) | p |
| **∆P_AW_ (cmH_2_O)** | 15.9 [15.1; 17.2] | 11.3 [9.9; 12.7] | **0.004** | 15.7 [12.2; 17.4] | 13.6 [10.7; 15.1] | ns |
| **Plateau P_AW_ (cmH_2_O)** | 25.6 [25.2; 26.9] | 25.8 [22.1; 28.9] | ns | 25.7 [24.4; 27.7] | 27.1 [22.2; 29.5] | ns |
| **PEEP (cmH_2_O)** | 9.3 [8.6; 10.0] | 15.0 [11.9; 16.3] | **0.03** | 10.5 [8.8; 12.7] | 12.2 [10.8; 14.7] | ns |
| **C_RS_ /kg** | 0.3 [0.3; 0.4] | 0.5 [0.4; 0.6] | ns | 0.4 [0.3; 0.5] | 0.4 [0.3; 0.6] | ns |
| **∆P_L_ (cmH_2_O)** | 14.9 [13.6; 16.1] | 9.2 [8.2; 10.6] | **0.002** | 13.5 [10.7; 16.3] | 11.0 [9.2; 13.7] | ns |
| **P_L-INS_ (cmH_2_O)** | 24.4 [23.1; 25.0] | 21.2 [17.9; 24] | ns | 23.4 [20.3; 25.2] | 23.4 [18.5; 25.1] | ns |
| **P_L_-_EXP_ (cmH_2_O)** | 1.1 [-0.2; 1.9] | -0.3 [-1.8; 1.7] | ns | 2.2 [0.7; 3.5] | 1.9 [-0.7; 4.2] | ns |
| **C_L_/kg** | 0.4 [0.3; 0.4] | 0.6 [0.5; 0.7] | **0.02** | 0.5 [0.4; 0.6] | 0.5 [0.4; 0.6] | ns |
| **C_CW_/kg** | 3.8 [2.8; 7.4] | 3.6 [3.4; 3.8] | ns | 4.1 [3.8; 4.5] | 3.6 [3.1; 4.9] | ns |
| **E_L_/E_RS_** | 0.90 [0.89; 0.95] | 0.83 [0.82; 0.83] | **0.002** | 0.89 [0.86; 0.93] | 0.87 [0.82; 0.92] | ns |
| **Vt/kg (mL/kg)** | 5.7 [4.8; 6.4] | 5.4 [5.1; 6.0] | ns | 6.3 [5.8; 6.9] | 5.1 [4.7; 5.8] | **0.03** |

Each available measure of respiratory mechanics was coupled to the simultaneous cardiac ultrasound. Data are presented in medians [IQR] and compared using Wilcoxon rank sum tests.
* Exclusion of infants (<1 year).
Abbreviations: ACP: acute cor pulmonale; C_CW_/kg: static compliance of the chest wall normalized per predicted body weight; C_L_/kg: static compliance of the lungs normalized per predicted body weight; C_RS_/kg: static compliance of the respiratory system normalized per predicted body weight; ∆P_AW_ : static airway driving pressure; ∆P_L_ : transpulmonary driving pressure; E_L_/E_RS_ : elastance ratio; P_L-INS_: end-inspiratory transpulmonary pressure (elastance-derived method); P_L-EXP_: end-expiratory transpulmonary pressure (direct method); Plateau P_AW_ : airway plateau pressure; PEEP: Positive end-expiratory airway pressure; Vt/kg: Tidal volume normalized per predicted body weight; RVD: Right ventricle dysfunction.

**Online figure 1.** Relationship between airway and transpulmonary driving pressures.


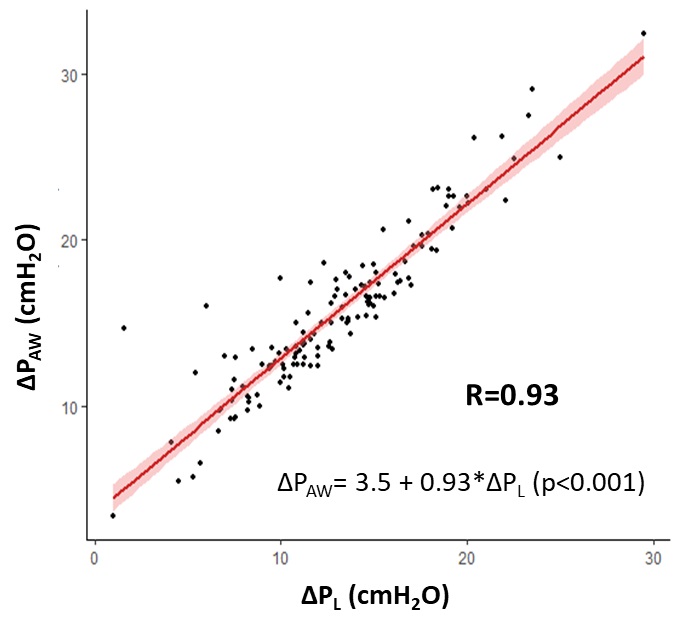


Correlation between driving airway pressure (ΔP_AW,_ Y axis) and transpulmonary driving pressure (ΔP_L,_ X axis) in all respiratory mechanics measurements. Each point represents one measure of respiratory mechanics, the solid red line represents the regression line and the colored red area represents the 95% confidence interval.
